# Maternal *GNAS* Contributes to the Extra-Large G Protein α-Subunit (XLαs) Expression in a Cell Type-Specific Manner

**DOI:** 10.3389/fgene.2021.680537

**Published:** 2021-06-17

**Authors:** Qiuxia Cui, Cagri Aksu, Birol Ay, Claire E. Remillard, Antonius Plagge, Mina Gardezi, Margaret Dunlap, Louis C. Gerstenfeld, Qing He, Murat Bastepe

**Affiliations:** ^1^Endocrine Unit, Department of Medicine, Massachusetts General Hospital and Harvard Medical School, Boston, MA, United States; ^2^Department of Thyroid and Breast Surgery, Zhongnan Hospital of Wuhan University, Wuhan, China; ^3^Department of Molecular Physiology and Cell Signalling, Institute of Systems, Molecular and Integrative Biology, University of Liverpool, Liverpool, United Kingdom; ^4^Department of Orthopaedic Surgery, Boston University School of Medicine, Boston, MA, United States; ^5^School of Stomatology, Wuhan University, Wuhan, China

**Keywords:** *GNAS*, stimulatory G protein, imprinting, osteoblasts, bone marrow stromal cells, fibrous dysplasia of bone

## Abstract

*GNAS* encodes the stimulatory G protein alpha-subunit (Gsα) and its large variant XLαs. Studies have suggested that XLαs is expressed exclusively paternally. Thus, XLαs deficiency is considered to be responsible for certain findings in patients with paternal *GNAS* mutations, such as pseudo-pseudohypoparathyroidism, and the phenotypes associated with maternal uniparental disomy of chromosome 20, which comprises *GNAS*. However, a study of bone marrow stromal cells (BMSC) suggested that XLαs could be biallelically expressed. Aberrant BMSC differentiation due to constitutively activating *GNAS* mutations affecting both Gsα and XLαs is the underlying pathology in fibrous dysplasia of bone. To investigate allelic XLαs expression, we employed next-generation sequencing and a polymorphism common to XLαs and Gsα, as well as A/B, another paternally expressed *GNAS* transcript. In mouse BMSCs, Gsα transcripts were 48.4 ± 0.3% paternal, while A/B was 99.8 ± 0.2% paternal. In contrast, XLαs expression varied among different samples, paternal contribution ranging from 43.0 to 99.9%. Sample-to-sample variation in paternal XLαs expression was also detected in bone (83.7–99.6%) and cerebellum (83.8 to 100%) but not in cultured calvarial osteoblasts (99.1 ± 0.1%). Osteoblastic differentiation of BMSCs shifted the paternal XLαs expression from 83.9 ± 1.5% at baseline to 97.2 ± 1.1%. In two human BMSC samples grown under osteoinductive conditions, XLαs expression was also predominantly monoallelic (91.3 or 99.6%). Thus, the maternal *GNAS* contributes significantly to XLαs expression in BMSCs but not osteoblasts. Altered XLαs activity may thus occur in certain cell types irrespective of the parental origin of a *GNAS* defect.

## Introduction

*GNAS* is an imprinted gene encoding the alpha-subunit of the stimulatory G protein (Gsα), a signaling protein mediating the actions of many endogenous molecules via generation of cAMP (Plagge et al., 2008; Weinstein et al., 2004; Mantovani et al., 2016). Loss-of-function mutations or epigenetic alterations of *GNAS* are responsible for different human diseases that involve abnormal skeletal development, metabolism, and hormone actions, such as pseudohypoparathyroidism Ia (MIM: 103580). Gain-of-function *GNAS* mutations affecting the activity of Gsα are found in a variety of benign and malignant tumors, as well as in patients with fibrous dysplasia of bone and McCune-Albright syndrome (MIM: 174800). *GNAS* gives rise to multiple additional products showing exclusively maternal or paternal expression (Bastepe, 2007; Kelsey, 2010). The extra-large G-alpha subunit (XLαs) is a variant of Gsα that uses a separate promoter and a unique first exon that splices onto Gsα exon 2 (Kehlenbach et al., 1994; Hayward et al., 1998). Hence, XLαs is partly identical to Gsα and can mimic the latter regarding cAMP generation (Klemke et al., 2000; Bastepe et al., 2002); however, XLαs mediates additional cellular actions, such as stimulation of IP3/PKC signaling and inhibition of clathrin-mediated endocytosis (He et al., 2015; He et al., 2017; He et al., 2019).

XLαs expression has been shown to occur exclusively from the paternal allele (Hayward et al., 1998), unlike Gsα, which is biallelic in most tissues. Thus, a *GNAS* mutation is predicted to affect XLαs only if it resides on the paternal allele. For example, the *GNAS* mutations causing pseudohypoparathyroidism type-1a are inherited maternally (Davies and Hughes, 1993) and, therefore, disrupt one copy of Gsα without affecting XLαs. In contrast, the same mutations in patients with pseudo-pseudohypoparathyroidism are inherited paternally and thus disrupt both XLαs and one copy of Gsα. The severe intrauterine growth retardation observed in pseudo-pseudohypoparathyroidism is attributed to XLαs deficiency (Richard et al., 2013). In addition, recent studies have described patients with maternal uniparental disomy of chromosome 20 (matUPD20, MIM: 617352)—the genomic region comprising *GNAS*—who display Silver-Russell syndrome-like features including perinatal growth retardation and intractable feeding difficulties (Mulchandani et al., 2016; Kawashima et al., 2018). The phenotypes associated with matUPD20 are similar to those observed in children with large paternal *GNAS* deletions (Aldred et al., 2002; Genevieve et al., 2005) and thus attributed largely to XLαs deficiency. Moreover, a recent study described an adult patient with hypophosphatemia and osteopetrosis who carried a nonsense mutation within the first XLαs exon (Chen et al., 2020). This defect was also paternally inherited, consistent with the evidence that XLαs is expressed from the paternal allele. Nevertheless, a study demonstrated XLαs expression from both alleles in human bone marrow stromal cells (BMSCs) from normal and fibrous dysplastic tissues (Michienzi et al., 2007). The analyzed BMSCs were clonogenic, and interestingly, Gsα expression was also shown to be allelically, but randomly, skewed, suggesting that the observed alterations in allelic XLαs and Gsα expression may have been secondary to the cloning.

BMSCs include mesenchymal progenitor/stem cells capable of differentiating into multiple different lineages. Gsα signaling promotes the commitment of mesenchymal progenitors into the osteoblast lineage while restricting osteoblast differentiation (Wu et al., 2011). Accordingly, a gain-of-function *GNAS* mutation results in the aberrant differentiation of BMSCs into osteoblasts, the primary cellular mechanism underlying fibrous dysplasia of bone (Xiao et al., 2019). The causative *GNAS* mutations are heterozygous and located in exons shared by Gsα and XLαs (Weinstein et al., 1991; Schwindinger et al., 1992). It has been shown that these mutations increase the baseline activity of not only Gsα but also XLαs (Klemke et al., 2000; He et al., 2015). Thus, whether XLαs is expressed monoallelically or biallelically in BMSCs could have strong implications for fibrous dysplasia pathogenesis. Moreover, as mentioned above, the phenotypes attributed to the loss of XLαs *in vivo* are based on the notion that XLαs is expressed in an exclusively paternal manner. If, however, tissue-specific biallelic XLαs expression exists, then our understanding of its physiological roles and its involvement in *GNAS*-related diseases may have to be revised.

In this study, we investigated the allele-specific expression of XLαs in non-clonal mouse and human BMSCs and other cells and tissues. We quantitated allelic contribution by employing next-generation sequencing (NGS) of RT-PCR amplicons carrying a polymorphism. Our results revealed a significant contribution from the maternal *GNAS* allele in non-differentiated BMSCs but not in osteogenically differentiated BMSCs or calvarial osteoblasts.

## Materials and Methods

### Mice

XLαs knockout (XLKO) mice, carrying a disruption in the first exon of XLαs, have been described (Plagge et al., 2004) and maintained in the CD1 background. Wildtype 129/Sv and C57BL/6 mice (Jackson Laboratory) were mated to obtain F1 generation hybrid offspring. Both male and female mice were analyzed. All mice experiments were conducted according to the accepted standards of the Institutional Animal Care and Use Committee and approved by the Massachusetts General Hospital Subcommittee on Research Animal Care.

### Restriction Digestion for Confirming Maternal and Paternal XLKO Samples and Determining the Parental Contribution of *Gnas* Transcripts

The methylation-sensitive restriction enzyme *Hpa*II (New England Biolabs) was used to digest the genomic DNA (1 μg) from tails of offspring derived from matings between heterozygous XLKO mice. Digested DNA was purified using the QIAquick PCR purification kit (Qiagen) and eluted with 30 μl water. Then, 2 μl of each sample was used as a template for genotyping PCR, as described (Plagge et al., 2004). To determine the parental contribution of Gsα, XLαs, and A/B transcripts, RT-PCR products from tissues of mice heterozygous for the C-to-G SNP in exon 11 (rs13460569) were incubated (37°C, 6 h) with *Ban*II (New England BioLabs) and analyzed by agarose gel electrophoresis, as described (Tafaj et al., 2017).

### Mouse Primary Cell Cultures and Osteogenic Differentiation

Mouse BMSCs were harvested by flushing the marrow out of long bones (femurs and tibias) of 14 week-old mice, as described (Wu et al., 2011). Cells were plated at a density of 0.5 × 10^6^ to 1 × 10^6^ cells/cm^2^ and cultured in Dulbecco’s Modified Eagle’s Medium (DMEM) (Gibco, United States) containing 10% Fetal Bovine Serum (FBS) (Hyclone, United States). Non-adherent cells were washed off, and the adherent cells were cultured for 1 week, followed by RNA extraction. Primary calvarial pre-osteoblasts were isolated at postnatal day five by serial collagenase digestion, as described (Mariot et al., 2011). Cells were seeded at a density of 10,000 cells/well in 12-well plates and cultured in Modified Eagle’s Medium (MEM) (Gibco, United States) containing 10% FBS. Osteoblastic differentiation was induced by adding ascorbic acid (50 μg/ml) and β-glycerol phosphate (10 mM) (Sigma-Aldrich), as described (Wu et al., 2011). Differentiation medium for BMSCs also included 10 nM dexamethasone (Sigma-Aldrich).

### Human BMSCs

Cells were obtained from participants who underwent total hip arthroplasty surgery at Boston Medical Center between 2019 and 2020. All human research was done under a Boston University School of Medicine Institutional Research Board Approved protocol: “Bone Tissues Repository,” IRB Number: H-35199, with patients’ consents per current HIPAA regulations prior to surgery and specimen collection. The femoral head and reamings, from the coring of the acetabulum, were collected during total hip arthroplasty. After multiple washes using DPBS (Hyclone Laboratories) containing an antibiotic-antimycotic mixture (Thermo Fischer Scientific) cells were suspended in DPBS. 24 million cells/well were seeded in each well of 6-well plates (Corning Inc.), treated with Animal Component-Free Cell Attachment Substrate (Stem Cell Technologies) diluted 1:150 in DPBS. Cells were cultured in an incubator at 37°C, 5% CO_2_, and > 90% humidity. A half media change was performed on day 4 and a full media change on day six after plating. Cells were then grown in basal medium supplemented with osteoinductive factors (Stem Cell Technologies) for 21 days before RNA extraction.

### Total RNA Extraction, RT-PCR and qRT-PCR Amplification

Total RNA of mouse organs was extracted by using Trizol^®^ Reagent (Life Technologies) followed by the Qiagen RNeasy^®^ Plus Mini Kit. Total RNA of human BMSCs was extracted using 4 M Guanidine-HCl, 1% Triton X-100, 10 mM Tris HCl, 2 mM EDTA (pH 7.4). cDNA was synthesized using the New England Biolabs ProtoScript II First-strand cDNA synthesis kit.

Total RNA of calvarial osteoblasts and BMSC were isolated by using the Qiagen RNeasy^®^ Plus Mini kit. One μg total RNA was reverse transcribed using the Superscript III First-Strand Synthesis System (Invitrogen) and then subjected to PCR using the PCR Master Mix (Promega). PCR mixture contained 2 μL cDNA in a final volume of 20μL. PCR included 1 cycle at 94°C for 5 min, followed by 40 cycles of 94°C for 30 s, 56°C for 30 s and 72°C for 2 min, and a final extension at 72°C for 7 min. For XLαs and A/B, nested PCR was performed using, as template, the primary PCR product diluted 1:10 or 1:2, respectively. qRT-PCR included 1 cycle at 50°C for 2 min and 94°C for 15 min, followed by 50 cycles of 94°C for 15 s, 60°C for 20 s, and 72°C for 20 s. The PCR primers are listed in Supplementary Table 1.

### Sequence Analysis of RT-PCR Products

The RT-PCR products were purified by the QIAquick PCR Purification kit. Both Sanger sequencing and NGS was performed at the Massachusetts General Hospital DNA Core. Primers for Sanger sequencing were the same as PCR primers. For NGS, purified PCR products were first fragmented and then subjected to NGS library preparation, followed by sequencing on the Illumina MiSeq. The reads were analyzed following automated *de novo* sequence assembly. The authenticity of the amplicons was confirmed by the unique first exon sequences of XLαs and A/B. To determine the relative contributions of the parental alleles, the ratio of reads containing one of the nucleotides to the total number of reads at rs13460569 (mouse) or rs7121 (human) was calculated for each sample.

### Genomic DNA Methylation Analysis

Genomic DNA (600 ng) from non-differentiated and differentiated BMSCs (the same samples as those used for allelic XLαs expression) was treated with sodium bisulfite by using the EZ DNA Methylation gold kit (Zymo Research), and the converted DNA was recovered in 10 μl. Primary PCR was performed using 2 μl bisulfite-treated DNA as template in a total reaction volume of 40 μl. The nested PCR used 2 μl of the primary PCR product. PCR included initial denaturation at 95°C for 5 min, 35 cycles of 95°C for 30 s, 56°C for 30 s, 72°C for 30 s, and final extension at 72°C for 10 min. Primers for the region within XLαs exon were previously described (Liu et al., 2000). Primers for the XLαs promoter were as follows: 5′-GATGGGGAGGGAGGTTTTTA-3′ (forward) and 5′–AAAACTAAAACCCAAAACCATAACT-3′ (reverse). Nested PCR used the same forward primer together with the following reverse primer: 5′-TCACCTTCCTAATTACACTTACCC-3′. The amplicons were subjected to NGS at the Massachusetts General Hospital DNA Core. To calculate the degree of methylation, paired-end NGS reads were aligned using the BWA-MEM algorithm to a reference reflecting bisulfite-induced alterations except for the CpG dinucleotides. The FreeBayes variant detector was then used to identify each C-to-T variant and the allelic balance (i.e., C is methylated and T non-methylated). BWA-MEM and FreeBayes were run on the Galaxy platform^1^ (Afgan et al., 2018).

### Statistical Analyses

Outliers were identified by the Grubbs test and excluded. One-way ANOVA followed by Tukey’s *post-hoc* test was used to analyze the differences among three or more groups. The student’s *t*-test (two-tailed) was used between two groups. The paired Student’s *t*-test (two-tailed) was employed for the differences between non-differentiated and differentiated BMSC samples. Wilcoxon rank-sum test was performed to compare the groups regarding the distribution of the data. One-sample *t*-test was performed to determine the significance of the difference between the maternal expression in non-differentiated BMSCs and the hypothetical value of 0%. *P* < 0.05 were considered significant. Analyses were performed using Prism 8 (GraphPad).

## Results

### Comparing XLαs mRNA Levels in Wildtype and XLKO Mice to Determine Allele-Specific XLαs Expression

**FIGURE 1:**
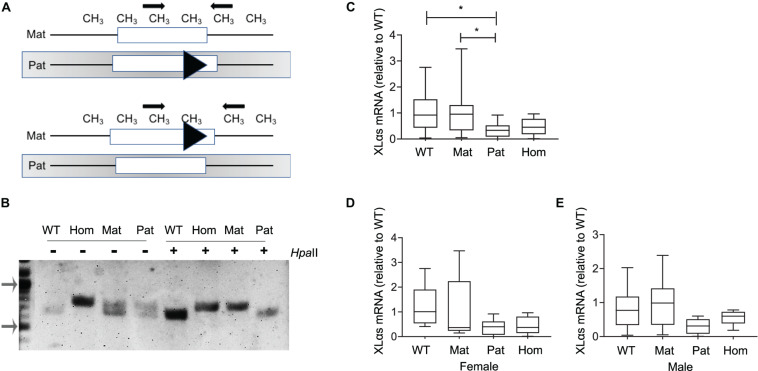
XLαs mRNA levels in maternal and paternal XLαs knockout mice to examine the allele-specific expression of XLαs. **(A)** Depiction of the normal and targeted (triangle) Gnas alleles comprising the first exon of XLαs in mice in which either the paternal (above) or the maternal (below) allele is targeted. The methylation (CH_3_)-sensitive restriction enzyme *Hpa*II cleaves the unmethylated (paternal) allele at multiple sites. Thus, the PCR performed using the *Hpa*II-digested DNA detects only the intact maternal allele. **(B)** PCR products from WT (wildtype), homozygous (hom), maternal (mat), and paternal (pat) DNA samples obtained before (−) or after (+) the *Hpa*II restriction enzyme. Note that the targeted allele produces a larger product. PCR products from maternal and homozygous XLKO after the *Hpa*II digest are larger than those from paternal XLKO and WT. Arrows indicate the 1,000- and 500-bp bands in the DNA ladder. **(C)** Box and whisker plots showing XLαs mRNA levels in bones from 14-week-old WT, maternal XLKO, paternal XLKO, and homozygous XLKO mice. **(D,E)** Female and male mice plotted separately. RNA was obtained from femurs after flushing out the bone marrow. qRT-PCR was performed with β-Actin as reference. Data were normalized to wildtype. *p < 0.05; using one-way ANOVA, followed by Tukey’s *post-hoc* test for pairwise comparisons; *n* = 18 WT (8 female and 10 male), 16 maternal XLKO (5 female and 11 male), 17 paternal XLKO (6 female and 11 male), and 16 homozygous XLKO (10 female and 6 male).

To examine the allelic expression profile of XLαs in bone, we first employed adult XLKO mice, because disruption of paternal XLαs first exon in those mice was reported to cause a dramatic reduction of XLαs transcript levels in various tissues (Plagge et al., 2004). The maternal, paternal, and homozygous XLKO mice were derived from matings between heterozygous XLKO mice. The previously described genotyping approach for these mice (Plagge et al., 2004) is unable to distinguish maternal from paternal XLKO mice. We thus performed genotyping before and after digestion of genomic DNA with the methylation-sensitive restriction enzyme *Hpa*II, which cleaves the unmethylated, paternal allele at multiple sites and, thereby, prevents it from serving as a template in PCR (Figure 1A). The PCR performed following the *Hpa*II digest detected only the intact maternal allele and allowed us to identify the parental origin of the targeted allele. In the absence of the *Hpa*II digestion, heterozygotes showed a double band, the upper one representing the targeted allele, and wildtype (WT) and homozygous XLKO mice yielded only the smaller or the larger product, respectively (Figure 1B). Following the *Hpa*II digest, only the smaller PCR product was detected in WT and paternal XLKO mice, and only the larger PCR product was observed in homozygous and maternal XLKO mice (Figure 1B).

qRT-PCR using total RNA from femur samples (bone marrow flushed out) showed that XLαs mRNA levels were significantly lower in paternal XLKO mice than in WT or maternal XLKO littermates (Figure 1C). While this finding was consistent with the silencing of the maternal allele in bone, other comparisons did not corroborate this conclusion. No statistically significant differences could be detected between the levels in homozygous XLKO mice and either WT or maternal XLKO (Figure 1C). Separate analysis of males and females did not reveal any significant differences between the groups, owing partly to the reduced sample sizes (Figures 1D,E).

### Intercrosses of C57BL/6 and 129/Sv Mice Allows Identification of Parental *Gnas* Alleles

**FIGURE 2:**
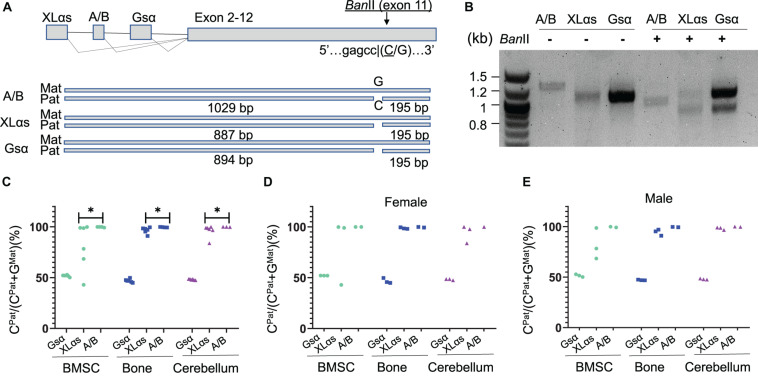
The use of an exon 11 C-to-G polymorphism reveals significant sample-to-sample variation in allelic XLαs expression. **(A)** Schematic presentation of XLαs, A/B, and Gsα transcripts in the mouse *Gnas* locus. The cytosine nucleotide in SNP rs13460569 introduces a *Ban*II restriction site. The intercrosses of C57BL/6 and 129/Sv mice were designed, such that the *Ban*II site is present on the paternal *Gnas* allele (i.e., inherited from the 129/Sv fathers). The expected sizes are depicted for each RT-PCR amplicon below. **(B)** Agarose gel electrophoresis of *Ban*II-digested and undigested RT-PCR amplicons, using total RNA of BMSCs from 14-week old F1 generation mice. First lane, DNA ladder. **(C–E)** The percentage of C-containing (paternal) NGS reads relative to the total number of reads. Complete data **(C)** and data from females **(D)** and males **(E)** are shown separately from individual mice. The RT-PCR products using total RNA from BMSCs, bone, and cerebellum of 14 week-old F1 generation mice were subjected to NGS. ^∗^*p* < 0.05 regarding the difference between the distribution of data points for XLαs and A/B (the Wilcoxon rank-sum test).

To determine allelic XLαs expression without relying on gene disruption, we then took advantage of a polymorphism located in *Gnas* exon 11, as described (Tafaj et al., 2017). The 129/Sv mice carry a cytosine (C), while C57BL/6 and CD1 strains carry a guanine (G) in the Gsα transcript (NM_201616.2, c.1009; rs13460569). We thus set up matings between 129/Sv males and C57BL/6 females and analyzed the offspring at age 14 weeks. RT-PCR using total RNA isolated from BMSCs amplified specific products from Gsα, XLαs, and A/B transcripts including the exon 11 polymorphism, and those products were then digested with *Ban*II, a restriction enzyme that recognizes the polymorphism and differentially cleaves the paternally-derived C-containing products (Figure 2A). *Ban*II partially cleaved the Gsα-derived products (Figure 2B), consistent with the previously demonstrated biallelic expression of this *Gnas* product in multiple tissues (Tafaj et al., 2017). In contrast, the A/B-derived products were completely cleaved, indicating that, consistent with previous observations (Tafaj et al., 2017), the A/B expression was nearly completely paternal (Figure 2B). A significant portion of the XLαs-derived RT-PCR products was cleaved; however, a faint band representing non-cleaved products were also visible, suggesting that XLαs expression may not be exclusively paternal in BMSCs (Figure 2B).

### NGS to Quantitate the Parental Origin of XLαs Expression

To have a quantitative assessment of XLαs expression from paternal vs. maternal *Gnas* alleles, we then subjected the RT-PCR products to NGS. Providing sequence data from thousands of molecules from each PCR amplicon, this approach revealed the origin of each read with respect to its parental origin, i.e., C-containing reads are derived from the paternal allele, while G-containing reads are from the maternal allele. We analyzed BMSCs and femur, as well as cerebellum, in which XLαs was shown to be expressed at high levels (Pasolli et al., 2000). As shown in Figures 2C–E and Supplementary Table 2, the Gsα products contained ∼50% C-containing products in all the tissues tested, thus confirming the biallelic transcription of Gsα. The C- containing reads in A/B transcript-derived PCR products were nearly 100% of the total in all three tissues, indicating exclusive paternal expression. In XLαs-derived RT-PCR products, however, the ratio of C-containing to G-containing reads varied significantly from sample to sample, particularly in BMSCs, ranging from 43.0 to 99.9% among six independent samples (Figures 2C–E and Supplementary Table 2). Modest sample-to-sample variation was also detectable in bone (83.7–99.6%) and cerebellum (83.8–100%; Figures 2C–E and Supplementary Table 2), and the variation observed in XLαs-derived products was significantly different from the variation observed in A/B-derived products in all three tissues.

### Allelic XLαs Expression in Differentiated Osteoblasts

**FIGURE 3:**
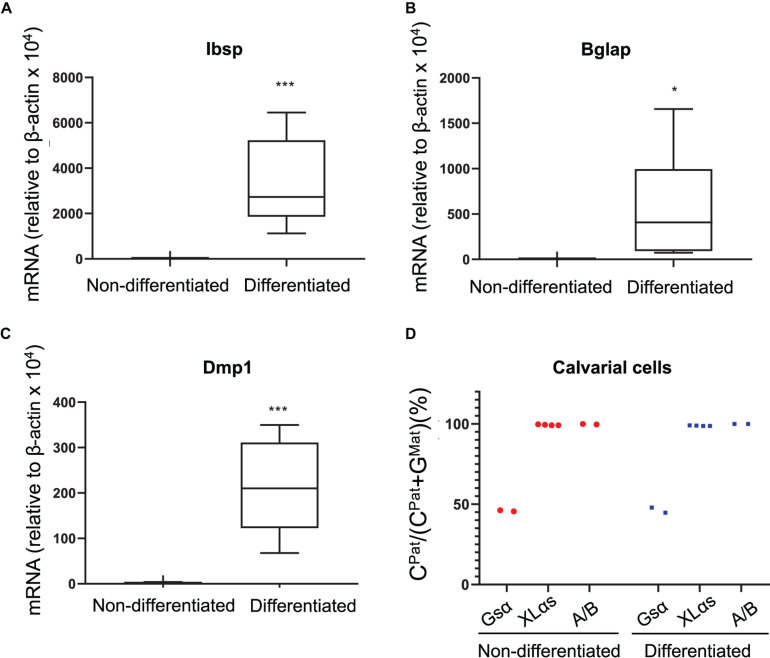
Allele-specific XLαs expression in cultured preosteoblasts and osteoblasts is almost exclusively paternal. The mRNA levels of osteoblast markers Ibsp **(A)**, Bglap **(B)**, and Dmp1 **(C)** were measured to confirm osteoblastic differentiation of calvarial preosteoblasts. qRT-PCR was used with β-actin as reference control. *p < 0.05; ***p < 0.001 vs. non-differentiated by two-tailed Student’s *t*-test (*n* = 7). **(D)** Percent of C-containing (paternal) NGS reads in RT-PCR amplicons from Gsα, XLαs, and A/B transcripts in non-differentiated and 14 day-differentiated preosteoblasts. Data are from individual F1 mice of C57BL/6 × 129/Sv intercrosses.

As another type of cultured primary cells, we examined mouse calvarial pre-osteoblasts from the C57BL/6-129/Sv intercrosses before and after culturing them under osteogenic conditions for 14 days. qRT-PCR experiments confirmed the osteoblast markers bone sialoprotein (Ibsp), osteocalcin (Bglap), and dentin matrix protein-1 (Dmp1) (Figures 3A–C). The NGS analysis of Gsα-derived RT-PCR products showed comparable numbers of C- and G-containing reads in both non-differentiated and differentiated cells, indicating biallelic expression (Figure 3D). In contrast, C-containing reads were nearly 100% of total reads for both XLαs and A/B products in those cells and there was no evidence of variation in the allelic XLαs expression (Figure 3D).

**FIGURE 4:**
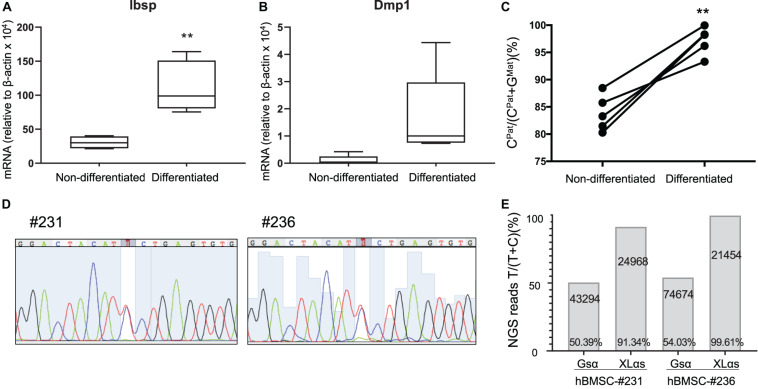
Allele-specific XLαs expression becomes increasingly paternal with osteoblastic differentiation of BMSCs. The mRNA levels of osteoblast markers Ibsp **(A)** and Dmp1 **(B)** were measured to confirm osteoblastic differentiation of BMSCs. qRT-PCR was used with β-actin as reference control. **p < 0.01 vs. non-differentiated by two-tailed Student’s *t*-test (*n* = 5). **(C)** Percent of C-containing (paternal) NGS reads in XLαs RT-PCR amplicons before and after differentiation toward the osteoblast lineage. Data are from individual F1 intercrosses of C57BL/6 × 129/Sv. **p < 0.01 vs. non-differentiated by paired Student’s *t*-test. **(D)** Sanger sequencing traces showing the heterozygous *GNAS* exon 5 C-to-T polymorphism in two separate human BMSC (hBMSC) samples. **(E)** Percentage of T-containing reads relative to total reads obtained from the NGS of Gsα and XLαs RT-PCR products. The total number of NGS reads for each amplicon is indicated. T-containing reads for #231-Gsα: 4017 (forward), 17727 (reverse); #231-XLαs: 1179 (forward), 21533 (reverse); #236-Gsα: 6522 (forward), 33294 (reverse); #236-XLαs: 922 (forward), and 20448 (reverse).

We then analyzed a new set of BMSCs both before and after osteogenic differentiation, which was confirmed by measuring the levels of Ibsp and Dmp1 mRNA (Figures 4A,B). In this new set of samples, while the paternal expression of A/B was nearly 100%, the paternal XLαs expression varied modestly from 80.3 to 88.5% of total (Figure 4C and Supplementary Table 3). This finding confirmed the significant contribution from the maternal allele to XLαs expression (mean = 16.1 ± 1.5%, *p* < 0.001 vs. the hypothetical 0% maternal). Combined, the mean maternal expression in all our BMSC samples (*n* = 11) was 17.6 ± 4.9% (95% confidence interval: 6.6–28.6%; *p* < 0.01 vs. the hypothetical value of 0%). Strikingly, when cells were grown under osteoinductive conditions for 2 weeks, the expression of XLαs shifted in the favor of the paternal allele, from 83.9 ± 1.5% paternal in non-differentiated to 97.2 ± 1.1% paternal in differentiated BMSCs (Figure 4C).

We also tested human BMSCs from two unrelated individuals, employing a C-to-T polymorphism located in *GNAS* exon 5 (rs7121, chr20:58903752, hg38). The samples (#231 and #236) were cultured under osteogenic conditions before examining the relative contribution of each allele to Gsα and XLαs expression. Sanger sequencing of Gsα-derived RT-PCR products revealed that both samples were heterozygous for the polymorphism (Figure 4D). Subsequent NGS of those products showed comparable numbers of reads containing either C or T at the polymorphic site, with T-containing reads making up 50.4 or 54.0% of total reads (Figure 4E). In contrast, NGS of XLαs-derived RT-PCR products yielded 91.3 and 99.6% T-containing reads in #231 and #236, respectively (Figure 4E), indicating predominantly or nearly exclusively monoallelic expression.

### Differential DNA Methylation at the Genomic XLαs Locus

**FIGURE 5:**
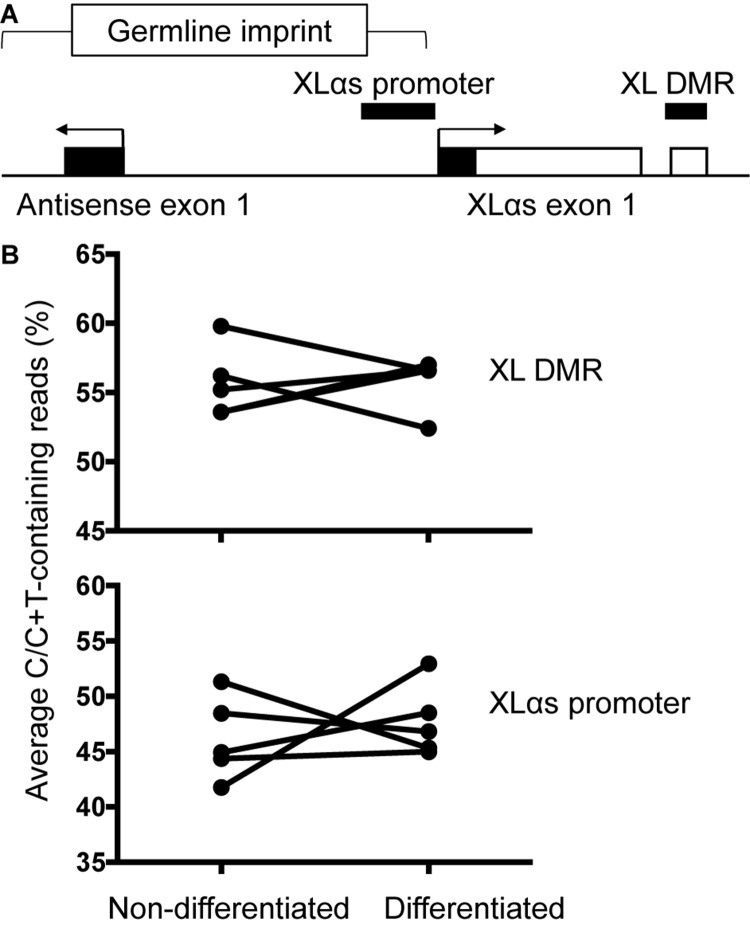
Methylation of XLαs promoter and DMR in mouse BMSCs. **(A)** The genomic region comprising XLαs exon 1 and the promoter, including exon 1 of the *GNAS* antisense (also known as Nespas). The analyzed regions are indicated by horizontal bars. The five CpGs analyzed at XL DMR span chr2:174300870-174300902 (mm 10) and the 30 CpGs analyzed at XLαs promoter span chr2:174297234-174297623 (mm 10). Exons (open: coding, closed: non-coding) and introns are depicted by boxes and connecting lines, respectively. The direction of transcription is indicated by arrows. The female germline imprint previously identified in this region is indicated. **(B)** Average methylation across the analyzed region in individual non-differentiated and osteogenically differentiated mouse BMSC samples. The differences are not statistically significant; *p* = 0.92 for the XLαs DMR and *p* = 0.62 for the XLαs promoter (paired *t*-test, 2-tailed).

The genomic region comprising the first XLαs exon is differentially methylated, consistent with the monoallelic expression of XLαs transcripts. We thus analyzed the degree of methylation in the previously described region (Liu et al., 2000). PCR amplicons derived from bisulfite-treated genomic DNA were subjected to NGS, thus allowing accurate relative quantification of unmethylated (converted to T) and methylated (protected) CpGs at multiple sites. The results, however, showed no significant differences in the overall degree of methylation between non-differentiated and differentiated BMSCs (average of 55.7 and 55.8% of total, respectively; Figures 5A,B). We also analyzed the methylation status of a second region, which overlaps the XLαs promoter (Williamson et al., 2006; Aydin et al., 2009; Figure 5A). The methylation of individual CpGs and the average methylation across the amplicon were comparable between non-differentiated and differentiated BMSCs (average 46.2 and 47.7%, respectively; Figure 5B and Supplementary Table 4).

## Discussion

In this study, we examined the parental contribution of *GNAS* allele to XLαs expression in non-clonal BMSCs and other murine tissues. In bone and cultured calvarial osteoblasts, XLαs expression was nearly exclusively paternal. In contrast, substantial maternal contribution was detected in non-differentiated BMSCs. The expression of Gsα was biallelic and that of A/B was virtually exclusively paternal in all the investigated cells/tissues.

To assess allelic XLαs expression, we first measured XLαs mRNA levels in paternal, maternal, and homozygous XLKO mice and their wildtype littermates. A similar approach has been used to measure the monoallelic expression of Gsα in certain tissues, such as proximal renal tubules (Turan et al., 2014). However, although the results appeared broadly consistent with an allelic bias of XLαs expression in bone, the mRNA levels varied widely, even within the same genotype. As this approach relied on the loss of XLαs production from the disrupted allele, this variability may reflect some detectable levels of XLαs mRNA being made from the latter allele, in addition to the outbred CD1 background in which XLKO mice had to be maintained (Plagge et al., 2004).

As an alternative approach, we utilized a known polymorphism in the mouse *Gnas* locus. We subjected the RT-PCR products to NGS, since it has been utilized for quantitatively detecting mosaic single nucleotide variants in clinical settings (Narumi et al., 2013). Indeed, NGS allowed us to obtain sequence information from thousands of reads and distinguish the allelic origin of the products. We analyzed the allele-specific XLαs and Gsα expression in human BMSCs similarly, using NGS and a common polymorphism (Campbell et al., 1994). In the human samples, genotypes of the donors’ parents were unknown. Therefore, although we could quantify the allele-specific expression, we were unable to determine the parental origin.

Michienzi et al. examined clonal BMSCs from wildtype and fibrous dysplasia-derived samples and showed evidence of biallelic XLαs expression, as well as parental Gsα expression that appeared asymmetrical (Michienzi et al., 2007). We analyzed non-clonal BMSCs passaged no more than three times. In total, we found biallelic XLαs expression in one sample and substantial deviation from monoallelic expression in seven samples, while the results from the remaining three samples were consistent with nearly exclusive paternal expression. There might be an influence of cell culture on our results. Nonetheless, no such variation was observed among our calvarial osteoblast samples, which were also cultured. Moreover, in BMSCs that were differentiated in culture toward the osteoblast lineage, the XLαs expression became more significantly paternal, arguing against the possibility that culture conditions caused a relaxation of XLαs imprinting.

A plausible explanation for the observed variation in allelic XLαs expression in our BMSC samples may be cellular heterogeneity, as BMSCs are known to be heterogeneous with variable representations of cell types in different samples (Kfoury and Scadden, 2015). Therefore, it is likely that XLαs is expressed biallelically in a certain subset of cells in the bone marrow, as well as in some other tissues. Regarding the study by Michienzi et al. (2007), the analyzed clonogenic BMSCs may have been derived from the cell-type(s) in which XLαs expression is biallelic. Alternatively, the maternal XLαs promoter may be active in all cell types in the BMSC samples but at a lower level than the paternal XLαs promoter. However, when considering the heterogeneity of BMSCs regarding cell types and the variation we observed among the samples, the former possibility appears more plausible. The finding that substantial XLαs expression occurs from the maternal allele in certain cell types has important implications. The physiological roles of XLαs *in vivo* have thus far been deduced from findings in mice or patients in whom the paternal XLαs allele is disrupted alone, assuming a complete loss of this protein in all tissues. According to our results, some of those interpretations could be at least partially incorrect.

We found paternal XLαs expression in mouse whole bones, although significant but modest sample-to-sample variation was observed in the parental contribution, as opposed to A/B expression, which appeared consistently ∼100% paternal. XLαs is substantially expressed in the cerebellum (Pasolli et al., 2000). We also detected mild variation in the allelic expression of XLαs among our mouse cerebellum samples, suggesting that in both bone and cerebellum, there may be a small subset of cells in which XLαs expression is biallelic.

Unlike the allelic expression data, we did not detect differences in DNA methylation between non-differentiated and differentiated BMSCs. The data for the methylation analysis refelected all the cells in the sample while the expression data originated only from cells that express XLαs. Since the methylation differences are expected to occur only in XLαs-expressing cells, they may have remained under our detection limit, particularly if those cells are scarce. Alternatively, other methylated regions or regulatory events may dictate the allelic shift of XLαs expression during osteogenic differentiation. The analyzed XLαs promoter region lies within a germline imprint including the *Gnas* antisense exon 1 (also known as Nespas; Coombes et al., 2003; see Figure 5A). It is possible that a different part of this germline imprint regulates the XLαs promoter activity. Also, a methylation-independent mechanism may be involved, considering that an “uncoupling” of allelic silencing and promoter DNA methylation has been described for the Nespas-mediated regulation of the Nesp55 promoter activity (Williamson et al., 2011). The regulation of allelic expression in this locus is certainly complex. It is also worth mentioning that the analyzed XLαs promoter region is orthologous to human *GNAS*-AS2, a region shown to be severely hypomethylated (i.e., loss of methylation) in patients with both sporadic and familial pseudohypoparathyroidism type-Ib (MIM: 603233) (Rochtus et al., 2016). Whether the hypomethylation is associated with biallelic XLαs expression is unknown.

Tissue- or cell type-specificity has been shown for the monoallelic expression of some transcripts, including Gsα, which is biallelic in most but predominantly maternal in some tissues (Yu et al., 1998). Conversely, Dlk1, a product of the imprinted gene cluster on chromosome 14, is paternally expressed nearly in all tissues but exhibits biallelic expression in postnatal neural stem cells and niche astrocytes (Ferron et al., 2011). Our observations regarding the allelic expression of XLαs are analogous to those documented for Dlk1 and supports the likelihood that XLαs is expressed biallelically in certain cell types. Identification of those cells may reveal important novel roles of XLαs.

In summary, our findings indicate that the maternal *GNAS* allele contributes significantly to XLαs expression in BMSCs. Thus, altered XLαs actions could be involved in at least some of the phenotypes associated with *GNAS* mutations even if the mutation is maternal.

## Data Availability Statement

The original contributions presented in the study are included in the article/Supplementary Material, further inquiries can be directed to the corresponding author/s.

## Ethics Statement

The studies involving human participants were reviewed and approved by the Boston University School of Medicine Institutional Research Board. The patients/participants provided their written informed consent to participate in this study. The animal study was reviewed and approved by Subcommittee on Research Animal Care, Massachusetts General Hospital. Written informed consent was obtained from the owners for the participation of their animals in this study.

## Author Contributions

QC: investigation, methodology, formal analysis, visualization, writing, and original draft. CA, BA, CR, MG, and MD: investigation. AP and LG: resources, writing–review, and editing. QH: investigation, methodology, writing—review, and editing. MB: conceptualization, formal analysis, funding acquisition, supervision, visualization, writing—original draft. All authors contributed to the article and approved the submitted version.

## Conflict of Interest

The authors declare that the research was conducted in the absence of any commercial or financial relationships that could be construed as a potential conflict of interest.

## Footnotes

1
https://usegalaxy.org/
