# Erratum: Maternal *GNAS* contributes to the extra-large G protein α-subunit (XLαs) expression in a cell type-specific manner

**DOI:** 10.3389/fgene.2024.1372003

**Published:** 2024-01-26

**Authors:** Frontiers Production Office

**Affiliations:** Frontiers Media SA, Lausanne, Switzerland

**Keywords:** *GNAS*, stimulatory G protein, imprinting, osteoblasts, bone marrow stromal cells, fibrous dysplasia of bone

Due to a production error, an **author name** was incorrectly spelled as “Quixia Cui”. The correct spelling is “Qiuxia Cui”.

The publisher apologizes for this mistake. The original version of this article has been updated.

