# Supplementary material for: Maternal GNAS Contributes to the Extra-Large G Protein α-Subunit (XLαs) Expression in a Cell Type-Specific Manner
Source: Front Genet. 2021 Jun 17;12:680537. doi: 10.3389/fgene.2021.680537 (PMC8247768; doi:10.3389/fgene.2021.680537)
Supplement: Supplementary file 1 [file Data_Sheet_1.docx]

**Supplementary Material**

**Maternal *GNAS* contributes to the extra-large G protein α-subunit (XLαs) expression in a cell type-specific manner**

Quixia Cui^1,2^, Cagri Aksu^1^, Birol Ay^1^, Claire E Remillard^1^, Antonius Plagge^3^, Mina Gardezi^4^, Margaret Dunlap^4^, Louis C Gerstenfeld^4^, Qing He^1,5^, and Murat Bastepe^1^*

1, Endocrine Unit, Department of Medicine, Massachusetts General Hospital and Harvard Medical School, Boston, MA

2, Department of Thyroid and Breast Surgery, Zhongnan Hospital of Wuhan University, Wuhan, China.

3, Department of Cellular and Molecular Physiology, Institute of Translational Medicine, University of Liverpool, Liverpool, UK

4, Department of Orthopaedic Surgery, Boston University School of Medicine, Boston, MA

5, School of Stomatology, Wuhan University, Wuhan, China

| **Table S1. RT-PCR and qRT-PCR primer sequences.** | |
| --- | --- |
| **Primer name** | **Sequence** |
| mXLαs-forward | 5'-CTCATCGACAAGCAACTGGA-3' |
| nested-mXLαs-forward | 5'-GAGAAGATGGACTACATGTG-3' |
| mA/B-forward | 5'-CGTCATCAGGCTGGTTAGAA-3' |
| nested-mA/B-forward | 5'-GTTGCTTCAGGTGGCTGGTA-3' |
| mGsα-forward | 5'-GCAGAAGGACAAGCAGGTCT-3' |
| mExon12-reverse | 5'-AGCTCGTATTGGCGGAGAT-3' |
| hGsα-forward | 5'-CCATGGGCTGCCTCGGGAACA-3' |
| hXLαs-forward | 5'-CGCAGTAAGCTCATCGACAA-3' |
| nested-hXLαs-forward | 5'-GGACGAAAAGATGGGCTACA-3' |
| hexon6-reverse | 5'-CCTTGGCATGCTCATAGAATTC-3' |
| mβ-actin-forward | 5'-GATCTGGCACCACACCTTCT-3' |
| mβ-actin-reverse | 5'-GGGGTGTTGAAGGTCTCAAA-3' |
| mXLαs-forward | 5'-CTCACTGACAAGCAACTGGA-3' |
| mExon2-reverse | 5'-CCCTCTCCGTTAAACCCATT-3' |
| mIbsp-forward | 5'-CAGAGGAGGCAAGCGTCACT-3' |
| mIbsp-reverse | 5'-CTGTCTGGGTGCCAACACTG-3' |
| mBglap-forward | 5'-CTTGAAGACCGCCTACAAAC-3' |
| mBglap-reverse | 5'-GCTGCTGTGACATCCATAC-3' |
| mDmp1-forward | 5'-CGCATCCCAATATGAAGACTG-3' |
| mDmp1-reverse | 5'-GCTTGACTTTCTTCTGATGACTCA-3' |

**Table S2. Numbers of C-containing (paternal) and G-containing (maternal) NGS reads obtained from the Gsα, XLαs, and A/B RT-PCR amplicons.**

|  |  | BMSC | | |  | Bone | | |  | Cerebellum | | |
| --- | --- | --- | --- | --- | --- | --- | --- | --- | --- | --- | --- | --- |
| # |  | C | G | C/(C+G) |  | C | G | C/(C+G) |  | C | G | C/(C+G) |
|  |  |  |  | (%) |  |  |  | (%) |  |  |  | (%) |
| 19 | Gsα | 5737 | 6224 | 48 |  | 5047 | 4249 | 54.3 |  | 2351 | 2126 | 52.5 |
|  |  |  | 670+ |  |  |  | 384+ |  |  |  | 183+ |  |
|  |  |  | 5554- |  |  |  | 3865- |  |  |  | 1943- |  |
|  | XLαs | 3567 | 4742 | 42.9 |  | 1178 | 18 | 98.5 |  | 3767 | 86 | 97.8 |
|  |  |  | 2345+ |  |  |  | 12+ |  |  |  | 77+ |  |
|  |  |  | 2389- |  |  |  | 6- |  |  |  | 9- |  |
|  | A/B | 7454 | 1 | 100 |  | 4783 | 30 | 99.4 |  | N.D. |  |  |
|  |  |  | 1+ |  |  |  | 25+ |  |  |  |  |  |
|  |  |  | 0 |  |  |  | 5- |  |  |  |  |  |
| 20 | Gsα | 1641 | 1781 | 48 |  | 3610 | 2946 | 55.1 |  | 4422 | 4197 | 51.3 |
|  |  |  | 199+ |  |  |  | 328+ |  |  |  | 464+ |  |
|  |  |  | 1582- |  |  |  | 2618- |  |  |  | 3733- |  |
|  | XLαs | 4604 | 42 | 99.1 |  | 3001 | 61 | 98 |  | 4138 | 799 | 83.8 |
|  |  |  | 31+ |  |  |  | 54+ |  |  |  | 608+ |  |
|  |  |  | 11- |  |  |  | 7- |  |  |  | 191- |  |
|  | A/B | N.D. |  |  |  | N.D. |  |  |  | N.D. |  |  |
| 21 | Gsα | 5020 | 5470 | 47.9 |  | 6618 | 6547 | 50.3 |  | 7079 | 6694 | 51.4 |
|  |  |  | 590+ |  |  |  | 550+ |  |  |  | 566- |  |
|  |  |  | 4880- |  |  |  | 5997- |  |  |  | 6128+ |  |
|  | XLαs | 10656 | 14 | 99.9 |  | 9824 | 44 | 99.6 |  | 6604 | 3 | 100 |
|  |  |  | 9+ |  |  |  | 41+ |  |  |  | 2+ |  |
|  |  |  | 5- |  |  |  | 3- |  |  |  | 1- |  |
|  | A/B | 6822 | 2 | 100 |  | 10696 | 4 | 100 |  | 9890 | 3 | 100 |
|  |  |  | 2+ |  |  |  | 4+ |  |  |  | 757+ |  |
|  |  |  | 0 |  |  |  | 0 |  |  |  | 6509- |  |
| 22 | Gsα | 2784 | 2967 | 48.4 |  | 6308 | 5722 | 52.4 |  | 7674 | 7275 | 51.3 |
|  |  |  | 249+ |  |  |  | 524+ |  |  |  | 604+ |  |
|  |  |  | 2718- |  |  |  | 5198- |  |  |  | 6671- |  |
|  | XLαs | 9488 | 4365 | 68.5 |  | 8196 | 1600 | 83.7 |  | 14746 | 124 | 99.2 |
|  |  |  | 3491+ |  |  |  | 1447+ |  |  |  | 113+ |  |
|  |  |  | 874- |  |  |  | 153- |  |  |  | 11- |  |
|  | A/B | 8510 | 3 | 100 |  | 5024 | 12 | 100 |  | 6402 | 0 | 100 |
|  |  |  | 3+ |  |  |  | 12+ |  |  |  | 0 |  |
|  |  |  | 0 |  |  |  | 0 |  |  |  | 0 |  |
| 62 | Gsα | 3901 | 4144 | 48.5 |  | 2980 | 2630 | 53.1 |  | 4194 | 3806 | 52.4 |
|  |  |  | 415+ |  |  |  | 229+ |  |  |  | 299+ |  |
|  |  |  | 3729- |  |  |  | 2401- |  |  |  | 3507- |  |
|  | XLαs | 5866 | 72 | 98.8 |  | 10202 | 497 | 95.3 |  | 3466 | 116 | 96.8 |
|  |  |  | 52+ |  |  |  | 435+ |  |  |  | 94+ |  |
|  |  |  | 20- |  |  |  | 62- |  |  |  | 22- |  |
|  | A/B | 8920 | 68 | 99.2 |  | 5356 | 30 | 99.4 |  | 1343 | 4 | 99.7 |
|  |  |  | 57+ |  |  |  | 23+ |  |  |  | 2+ |  |
|  |  |  | 11- |  |  |  | 7- |  |  |  | 2- |  |
| 63 | Gsα | 2187 | 2203 | 49.8 |  | 3686 | 3260 | 53.1 |  | 2353 | 2161 | 52.1 |
|  |  |  | 220+ |  |  |  | 341+ |  |  |  | 195+ |  |
|  |  |  | 1983- |  |  |  | 2919- |  |  |  | 1966- |  |
|  | XLαs | 6893 | 1903 | 78.4 |  | 8753 | 268 | 97 |  | 8716 | 100 | 98.9 |
|  |  |  | 1276+ |  |  |  | 233+ |  |  |  | 81+ |  |
|  |  |  | 624- |  |  |  | 35- |  |  |  | 19- |  |
|  | A/B | N.D. |  |  |  | N.D. |  |  |  | N.D. |  |  |

N.D. Not determined; All are 14-week old F1 generation mice from C57BL/6(G) x 129/Sv (C) intercrosses; All mice were born from the same breeding pair: 19-22 and 62, 63 were separate littermates. 19, 20, and 21 are females while 22, 62, and 63 are males.. RNAs were extracted from BMSC, femur, and cerebellum, reverse transcribed into cDNA, and then amplified by using primers for Gsα, XLαs, and A/B. Amplicons were subjected to next-generation sequencing (NGS). The percentage of C-containing (paternal) NGS reads relative to the total number of reads is shown. The number of G-containing (i.e. maternal) reads in the forward (+) and reverse (-) direction are shown below the total number.

**Table S3.** **Numbers of C-containing (paternal) and G-containing (maternal) NGS reads for XLαs RT-PCR amplicon from non-differentiated and differentiated BMSCs.**

|  |  | Non-differentiated | | | | | | |  | Differentiated | | |
| --- | --- | --- | --- | --- | --- | --- | --- | --- | --- | --- | --- | --- |
|  |  | A/B | | |  | XLαs | | |  | XLαs | | |
| # |  | C | G | C/(C+G) |  | C | G | C/(C+G) |  | C | G | C/(C+G) |
|  |  |  |  | (%) |  |  |  | (%) |  |  |  |  |
| 81 |  | 6735 | 2 | 100.0 |  | 11465 | 1905 | 85.8 |  | 6165 | 443 | 93.3 |
|  |  |  | 2+ |  |  |  | 964+ |  |  |  | 235+ |  |
|  |  |  | 0 |  |  |  | 941- |  |  |  | 208- |  |
| 82 |  | 437 | 5 | 98.9 |  | 10175 | 2313 | 81.5 |  | 7911 | 137 | 98.3 |
|  |  |  | 1+ |  |  |  | 1081+ |  |  |  | 78+ |  |
|  |  |  | 4- |  |  |  | 1232- |  |  |  | 59- |  |
| 83 |  | 5655 | 2 | 100.0 |  | 6577 | 1320 | 83.3 |  | 7936 | 314 | 96.2 |
|  |  |  | 2+ |  |  |  | 581+ |  |  |  | 180+ |  |
|  |  |  | 0 |  |  |  | 739- |  |  |  | 134- |  |
| 84 |  | 5718 | 5 | 99.9 |  | 8860 | 1155 | 88.5 |  | 5615 | 2 | 100.0 |
|  |  |  | 4+ |  |  |  | 531+ |  |  |  | 1+ |  |
|  |  |  | 1- |  |  |  | 624- |  |  |  | 1- |  |
| 85 |  | 6555 | 5 | 99.9 |  | 7734 | 1899 | 80.3 |  | 8658 | 156 | 98.2 |
|  |  |  | 2+ |  |  |  | 1046+ |  |  |  | 89+ |  |
|  |  |  | 3- |  |  |  | 853- |  |  |  | 67- |  |

All are 14-week old F1 generation mice from C57BL/6(G) x 129/Sv (C) intercrosses. All mice were female littermates from the same breeding couple (the male breeder was the same as the one for the mice detailed in Table S2). RNAs were extracted from cultured BMSCs, reverse transcribed into cDNA, and then amplified by using primers for XLαs and A/B. Amplicons were subjected to next-generation sequencing (NGS). The percentage of C-containing (paternal) NGS reads relative to the total number of reads is shown. The number of G-containing (i.e. maternal) reads in the forward (+) and reverse (-) direction are shown below the total number.

**Table S4. Methylation at individual CpGs of the mouse XLαs promoter region.**

|  | Differentiated - (%) C/T+C (METHYLATED) | | | | | |  | Non-differentiated - (%) C/T+C (METHYLATED) | | | | | |
| --- | --- | --- | --- | --- | --- | --- | --- | --- | --- | --- | --- | --- | --- |
| CpG Position* | D81 | D82 | D83 | D84 | D85 | Average per position | *P* (Paired t-test) | U81 | U82 | U83 | U84 | U85 | Average per position |
| 22 | 53.4 | 53.6 | 51.2 | 60.3 | 55.2 | 54.7 | 0.4 | 56.7 | 53.5 | 50.7 | 48.9 | 51.9 | 52.3 |
| 50 | 51.9 | 54.6 | 51.4 | 60.1 | 55.6 | 54.7 | 0.3 | 53.5 | 53.7 | 51.1 | 49.0 | 51.1 | 51.7 |
| 57 | 56.1 | 58.3 | 55.1 | 62.5 | 54.8 | 57.4 | 0.4 | 57.8 | 57.0 | 54.6 | 52.3 | 55.5 | 55.4 |
| 62 | 48.9 | 53.0 | 50.1 | 57.3 | 51.3 | 52.1 | 0.5 | 51.8 | 52.3 | 49.6 | 46.4 | 50.8 | 50.2 |
| 75 | 51.7 | 55.3 | 53.1 | 58.3 | 56.1 | 54.9 | 0.4 | 56.9 | 54.6 | 50.2 | 48.7 | 52.0 | 52.5 |
| 87 and 91 | 56.3 | 57.6 | 58.5 | 62.1 | 58.0 | 58.5 | 0.2 | 59.2 | 57.3 | 53.5 | 51.7 | 54.0 | 55.1 |
| 113 | 51.6 | 56.7 | 53.6 | 60.6 | 53.6 | 55.2 | 0.3 | 57.4 | 54.1 | 49.2 | 48.1 | 49.8 | 51.7 |
| 128 | 55.4 | 57.6 | 53.0 | 60.8 | 55.5 | 56.5 | 0.4 | 58.3 | 56.7 | 52.0 | 49.7 | 53.5 | 54.0 |
| 139 | 54.7 | 55.8 | 54.7 | 62.3 | 59.2 | 57.3 | 0.5 | 61.7 | 56.0 | 53.5 | 50.0 | 54.3 | 55.1 |
| 154 | 51.0 | 50.2 | 49.8 | 57.8 | 53.9 | 52.5 | 0.6 | 54.5 | 55.2 | 49.0 | 46.9 | 48.5 | 50.8 |
| 160 and 164 | 46.8 | 46.5 | 47.2 | 52.8 | 51.5 | 49.0 | 0.8 | 54.3 | 51.4 | 46.3 | 42.2 | 47.1 | 48.3 |
| 181 | 46.4 | 47.2 | 44.1 | 53.5 | 49.1 | 48.0 | 0.6 | 50.2 | 50.2 | 44.1 | 40.3 | 45.4 | 46.1 |
| 207 | 44.6 | 44.0 | 43.0 | 50.5 | 47.1 | 45.8 | 0.6 | 49.6 | 46.0 | 42.7 | 39.3 | 43.6 | 44.2 |
| 217 | 45.1 | 44.7 | 44.5 | 49.9 | 47.2 | 46.3 | 0.7 | 50.5 | 46.8 | 43.9 | 40.2 | 44.7 | 45.2 |
| 223 | 42.7 | 43.2 | 44.0 | 50.0 | 46.7 | 45.3 | 0.6 | 48.3 | 45.8 | 42.2 | 38.4 | 43.8 | 43.7 |
| 237 | 33.5 | 35.1 | 35.4 | 41.1 | 38.3 | 36.7 | 0.9 | 44.6 | 40.7 | 33.9 | 32.4 | 35.2 | 37.3 |
| 243 | 29.9 | 33.7 | 32.3 | 41.5 | 36.8 | 34.8 | 0.9 | 41.6 | 39.8 | 32.8 | 30.2 | 33.2 | 35.5 |
| 263 | 31.2 | 30.0 | 29.8 | 37.9 | 34.7 | 32.7 | 1.0 | 37.4 | 37.2 | 30.2 | 28.1 | 31.7 | 32.9 |
| 273 | 31.8 | 34.2 | 33.4 | 40.3 | 36.9 | 35.3 | 0.8 | 38.1 | 38.8 | 33.0 | 30.1 | 33.5 | 34.7 |
| 296 | 37.9 | 41.5 | 40.2 | 49.8 | 43.6 | 42.6 | 0.8 | 48.4 | 42.8 | 39.5 | 36.9 | 39.1 | 41.3 |
| 303 | 40.1 | 41.2 | 38.7 | 49.2 | 44.8 | 42.8 | 0.7 | 48.2 | 43.2 | 39.2 | 37.5 | 39.4 | 41.5 |
| 308 | 41.2 | 43.0 | 40.0 | 49.0 | 46.1 | 43.9 | 0.9 | 50.3 | 45.2 | 41.9 | 38.5 | 40.4 | 43.3 |
| 313 | 43.1 | 43.9 | 41.3 | 51.5 | 45.7 | 45.1 | 0.8 | 51.4 | 46.6 | 42.0 | 39.6 | 41.3 | 44.2 |
| 318 | 42.8 | 45.2 | 40.9 | 51.2 | 46.8 | 45.4 | 0.7 | 50.4 | 46.5 | 42.1 | 39.3 | 41.7 | 44.0 |
| 339 | 44.0 | 43.6 | 45.1 | 52.5 | 46.1 | 46.3 | 0.6 | 51.8 | 44.5 | 42.0 | 40.0 | 42.7 | 44.2 |
| 362 | 46.6 | 48.5 | 43.2 | 53.0 | 46.5 | 47.5 | 0.7 | 51.8 | 48.1 | 44.5 | 42.4 | 44.8 | 46.3 |
| 398 | 44.6 | 45.5 | 42.1 | 53.2 | 48.6 | 46.8 | 0.6 | 50.6 | 46.9 | 43.3 | 40.2 | 44.6 | 45.1 |
| 409 | 45.7 | 47.1 | 44.0 | 53.6 | 48.5 | 47.8 | 0.6 | 51.7 | 46.7 | 45.3 | 42.2 | 44.7 | 46.1 |
| Average | 45.3 | 46.8 | 45.0 | 52.9 | 48.5 | 47.7 | 0.6 | 51.3 | 48.5 | 44.4 | 41.8 | 44.9 | 46.2 |

* The CpGs span from chr2:174297234 – 174297623 (mm10). The positions are relative to the PCR amplicon analyzed by NGS.
